# Isolation and Characterization a Novel Catabolic Gene Cluster Involved in Chlorobenzene Degradation in Haloalkaliphilic *Alcanivorax* sp. HA03

**DOI:** 10.3390/biology11050724

**Published:** 2022-05-09

**Authors:** Mousa A. Alghuthaymi, Ahmed M. Awad, Hamdy A. Hassan

**Affiliations:** 1 Biology Department, Science and Humanities College, Shaqra University, Alquwayiyah 11726, Saudi Arabia; malghuthaymi@su.edu.sa; 2 Department of Environmental Biotechnology, Genetic Engineering and Biotechnology Research Institute, University of Sadat City, Sadat City 32897, Egypt; hamdy.hassan@gebri.usc.edu.eg

**Keywords:** chlorobenzene, chlorocatechol 1,2-dioxygenase, chlorobenzene dioxygenase, haloalkaliphilies

## Abstract

**Simple Summary:**

This study has demonstrated for the first time the metabolic pathway of chlorobenzene (CB) in haloalkaliphilic bacteria *Alcanivorax* sp. HA03 isolated from soda lakes in Wadi E1Natrun-Egypt. The complete chlorobenzene dioxygenase gene cluster (α and β subunits, ferredoxin and ferredoxin reductase) was detected by PCR amplification and confirmed by DNA sequencing and expression. This gene cluster has the capability to ring-hydroxylating CB transformed into 3-chlorocatechol during the first steps of biodegradation; with further chloride release and subsequent paths, HA03 showed capability for CB mineralization.

**Abstract:**

Chlorobenzene (CB) poses a serious risk to human health and the environment, and because of its low degradation rate by microorganisms, it persists in the environment. Some bacterial strains can use CB as growth substrates and their degradative pathways have evolved; very little is known about these pathways and the enzymes for CB degradation in high pH and salinity environments. *Alcanivorax* sp. HA03 was isolated from the extremely saline and alkaline site. HA03 has the capability to degrade benzene, toluene and chlorobenzene (CB). CB catabolic genes were isolated from HA03, which have a complete gene cluster comprising α and β subunits, ferredoxin and ferredoxin reductase (CBA1A2A3A4), as well as one gene-encoding enzyme for chlorocatechol 1,2-dioxygenase (CC12DOs). Based on the deduced amino acid sequence homology, the gene cluster was thought to be responsible for the upper and lower catabolic pathways of CB degradation. The CBA1A2A3A4 genes probably encoding a chlorobenzene dioxygenase was confirmed by expression during the growth on CB by RT-PCR. Heterologous expression revealed that CBA1A2A3A4 exhibited activity for CB transformation into 3-chlorocatechol, while CC12DOs catalyze 3-chlorocatechol, transforming it into 2-chloromucounate. SDS-PAGE analysis indicated that the sizes of CbA1 and (CC12DOs) gene products were 51.8, 27.5 kDa, respectively. Thus, *Alcanivorax* sp. HA03 constitutes the first bacterial strain described in the metabolic pathway of CB degradation under high pH and salinity conditions. This finding may have obvious potential for the bioremediation of CB in both highly saline and alkaline contaminated sites.

## 1. Introduction

Chlorobenzene (CB) is toxic, carcinogenic even in traces and listed as a priority pollutant [1]. Although CB is rarely formed naturally, enormous amounts deliberately or accidentally released into the biosphere through human activities for various manufacture purposes such as paints, dyes, industrial solvents, pesticides and insecticides, resulting in the accumulation of this compound in the food chain [2]. CB persists in the environment and its degradation is slow in comparison with other non-chlorinated aromatic compounds [3,4].

The biodegradation of CB has been described in several aerobic bacterial strains [5,6,7,8,9,10,11,12]. Very little information has been reported on the aerobic bacterial degradation of benzene and its chlorinated derivatives in extreme environments, such as low temperatures and hyper saline and alkaline conditions [13,14,15,16,17].

All the reported literature concerning the pathways and enzymes involved in the aerobic degradation of CB were for non-haloalkaliphilic bacteria [11,12,18]. The metabolic pathways of CB biodegradation by aerobic bacteria usually proceeds via chlorocatechol formation, which is the bottleneck in complete CB mineralization and mostly further degraded by the *ortho*-pathway and a modified *ortho*-pathway, and in some cases by the *meta*-cleavage pathway [11,12,19,20,21].

In the *ortho*-cleavage pathway, the lower metabolic pathway of CB by *ortho*-cleavage pathway starts by intradiol ring cleavage of chlorocatechol by chlorocatechol 1, 2-dioxygenase transformed into chloromuconate, which is subjected to subsequent enzyme chloromuconate cycloisomerase. This enzyme is capable of cycloisomerization and dehalogenation, forming *trans*-dienelactone [6,19,22].

There is no information in the literature to date about the pathways and enzymes for CB degradation in high pH and salinity. A few recent studies have described the pathways and enzymes involved in the benzene, toluene, ethylbenzene and xylenes (BTEX) degradation in extreme conditions, either high pH or high salinity, which have high similarity with enzymes described for many non-extremophiles [13,14,15,16,17].

However, there is a gap in the knowledge of the metabolic pathways of CB by haloalkaliphiles; the present study tries to elucidate the metabolic pathway of CB degradation for the first time in haloalkaliphilic bacteria *Alcanivorax* sp. HA03, and this could be important to develop and optimize CB bioremediation in high salinity contaminated sites.

## 2. Materials and Methods

### 2.1. Culture Conditions and Preparation of Cell Extracts from Alcanivorax *sp.* HA03

CB-degrading organism *Alcanivorax* sp. HA03 was previously isolated from Soda lakes from Wadi El Natrun-Egypt, which has high pH and salinity [14]. The best conditions for the degradation of CB by HA03 were on mineral media [23] supplemented with 3% NaCl (*w/v*) and pH 9 adjusted by NaHCO3 solution using 20 µmol of CB, as previously described [14]. Fluted Erlenmeyer flasks were used for growing HA03 cells and were incubated at 30 °C with shaking at 150 rpm. Using a spectrophotometer at 600 nm, the HA03 growth was monitored; the cells were harvested by centrifugation, washed and resuspended in mineral media. Cell extracts from HA03 were prepared [24] and determined [25]. Sodium dodecyl sulfate–12.5% polyacrylamide gel electrophoresis was applied for the supernatant fluids [26]. Moreover, 1% (*w/v*) Coomassie brilliant blue G-250 was used for staining the protein gels [27]. The amount of loaded protein after dilution could range from 1 to 30 µg. The denaturated protein subunits was evaluated for its molecular weight using the PageRulerTM Prestained protein ladder (Thermo Scientific, Waltham, MA, USA).

### 2.2. PCR Amplification of Chlorobenzene Dioxygenase Encoding Genes from HA03

The genomic DNA was extracted from the pure culture from HA03 strain using a GeneJET Genomic DNA Purification Kit (Thermo Scientifc). FHDO_forward 5′gcgatggaagaggttggtat 3′ and RHDO_ reverse 5′-cgcgccacctgttatcaat 3′ primer sets were designed from the alignment of the α subunit and ferredoxin reductase-conserved regions and residues are shared among all protein segments from the starting amino acids from different oxygenases described as initial dioxygenases for chloroaromatics.

### 2.3. Extraction of mRNA and cDNA Synthesis and RT-PCR

To assess the gene expression and constitutive expression, *Alcanivorax* sp. HA03 was grown on CB (1 mM) and in parallel on fructose (1 mM) as the sole carbon source. Total RNA was isolated from 3 mL of CB- or fructose-grown cells during the exponential growth. The harvested cells either from CB- or fructose-grown cells were resuspended in 100 µL of water and processed for RNA isolation [28,29,30]. After purification of RNA using the RNeasy kit (Qiagen), the eluted RNA was separated using 2 µL in 1% agarose gels and stained with ethidium bromide. RevertAid First Strand cDNA Synthesis Kit (Thermo Scientific) were used for cDNA synthesis using1 µL from RNA templates. Because of the maintained activity of M-MuLV Reverse Transcriptase (RT) at 42–50 °C, the kit uses RevertAid (RT). The supplied RiboLock RNase Inhibitor with the kit protected the RNA template from degradation. Serial 3.2-fold dilution from the reverse transcription reaction mixtures was applied using nuclease-free water (Qiagen). PCR was applied using 1 µL of each cDNA dilution, with primer set FBphARNA (5′-GTGCGGCAGAAAGATAAAGC-3′ and RBphARNA 5′-GTCCCCGGTTTTAGGAATGT-3′ targeting the α subunit (CBA1), in order to amplify the CBA1 gene fragment. The amplified product bands in 1% agarose gels were purified and sequenced to verify their identity.

### 2.4. Amplification and Characterization of Chlorocatechol 1,2 Dioxygenase

Chlorocatechol 1,2-dioxygenase (CC12Dos) was amplified from *Alcanivorax* sp. HA03. A primer set of degenerated primers, fw-cldioxmt (5′-GTITGGCAYTCIACICCIGAYGG-3′) and rev-cldioxmt (5′-CCICCCTCGAARTACTGIGT-3′), which had previously been designed for the amplification of ~280 bp fragment from chlorocatechol 1,2-dioxygenase, were involved in the degradation of chlorobenzene using touch down PCR [3]. In total, ~280 bp was ligated and transformed using the pGEM^®^-T Easy Vector and *E. coli* JM109.

### 2.5. Cloning and Transformation of Chlorobenzene Dioxygenase from HA03

Chlorobenzene dioxygenase gene cluster comprising the CBA1A2A3A4 was amplified using a primer set including artificial restriction sites FHDOPST1_forward (5′gcgatggaa**ctgcag**gttggtat-3′)/RHDOECO_reverse (5′-cgcgccacct**gaattc**atcaat 3′) for restriction enzymes *EcoR*1 and *Pst*1. A 4.5 kb DNA fragment containing the gene region comprising the CBA1A2A3A4 genes from HA03 was digested and cloned into pUC119 to give pCB and transformed into *E. coli* JM109. Different primer sets (FHDOPST1_forward/RHDOECO_ reverse) were used for amplification complete chlorobenzene dioxygenase encoding gene cluster α and β subunits, ferredoxin and ferredoxin reductase. *E. coli* JM109 was grown either in the presence or in the absence of IPTG. The depletion of CB was monitored by HPLC analysis [31]. To analyze if the CC12Dos encode functional chlorocatechol 1,2-dioxygenase, CC12Dos gene probably encoding a chlorocatechol 1,2-dioxygenase was amplified from HA03 using a CC1,2HF/CC1,2HR primer set, cloned into the TA cloning kit pGEM^®^-T Easy Vector (Promega) and transformed by using heat shock in *E. coli* JM109. *E. coli* JM109, harboring only vector without insert, was applied as control. Screening of white colonies was done with colony PCR for half of the colony, while the other was left to reculture. The same primer sets CC1,2HF and CC1,2HR were used for the amplification. The white colonies that harbored the inserted CC12Dos gene were recultured from the other half, and grown with IPTG, where the transformation of 200 μM from 3-Chlorocatechol was tested by HPLC using the resting cells of an OD600 nm [31].

### 2.6. Nucleotide Sequence Accession Numbers

The chlorobenzene dioxygenase and chlorocatechol 1,2-dioxygenase reported in this study are available under accession numbers JQ015309 and JQ687410 in GenBank.

## 3. Results

### 3.1. Amplification and Characterization the Initial Dioxygenase in Alcanivorax *sp.* HA03

**Figure 1:**
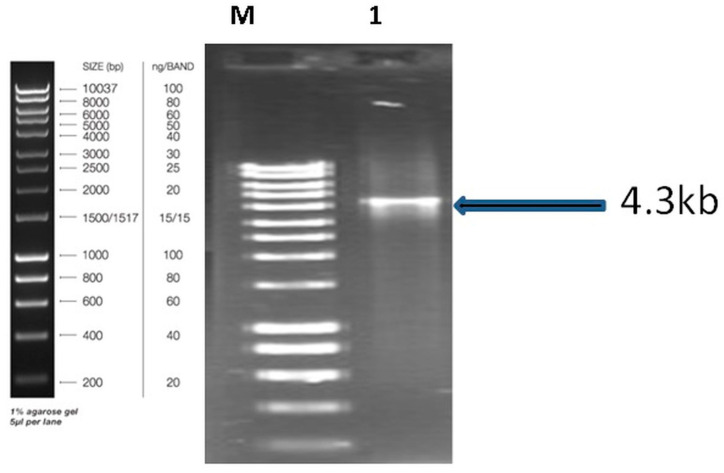
Lane 1 ~4.5 kb fragment was obtained using the FHDOPST_Forward/RHDOECO_ reverse primer set on genomic DNA of *Alcanivorax* sp. HA03 (lane 1). Lane M, molecular weight Hyperladder I (Bioline).

**Figure 2:**
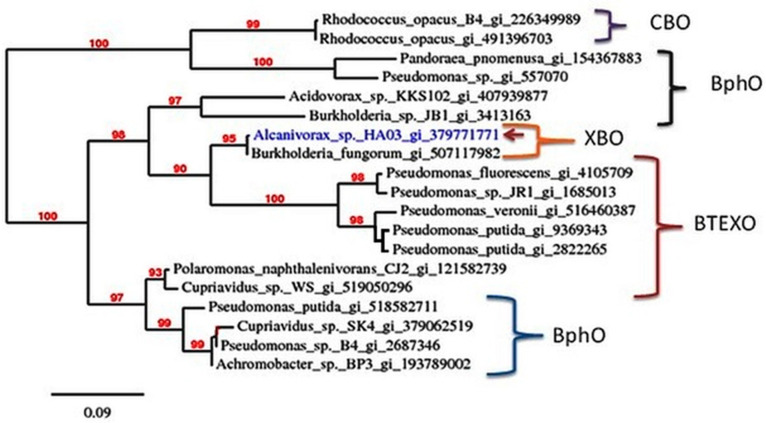
Phylogenetic showing the relatedness of the Alpha subunits of Rieske non-heme iron oxygenases of *Alcanivorax* sp. HAO3 with the other nearest Alpha subunits. The phylogenetic tree was generated using Phylogeny.fr based on protein sequence alignment. CBO (chlorobenzene dioxygenase); BphO (Biphenyl dioxygenase); XBO (halobenzene dioxygenase); BTEXO (Benzene, Toluene, Ethylbenzene and xylene dioxygenase).

As described, HA03 was able to utilize CB as a sole carbon source [13]. Very few genes involved in the metabolism of Benzene and its derivatives under haloalkaliphilic conditions have been described, and to the best of our knowledge, no study has been conducted to elucidate the metabolism of CB by haloalkaliphiles. CBA1A2A3A4 genes probably encode the complete initial CB dioxygenase α and β subunits with the electron transformation ferredoxin and ferredoxin reductase was amplified with ~4.5 kb (Figure 1) from HA03 using the primer set FHDO_forward/RHDO_reverse. In this study, chlorobenzene dioxygenase was successfully localized in HA03, where CBA1 exhibits 99% similarity with α subunit from halobenzene alkylbenzene dioxygenase from *Burkholderia fungorum* flu100 [32,33], 92% similarity with the dioxygenase from BTEX degraders *Pseudomonas veronii* and *Pseudomonas putida* 01G3 [34] and 82% similarity with biphenyl dioxygenase (Figure 2).

**Figure 3:**
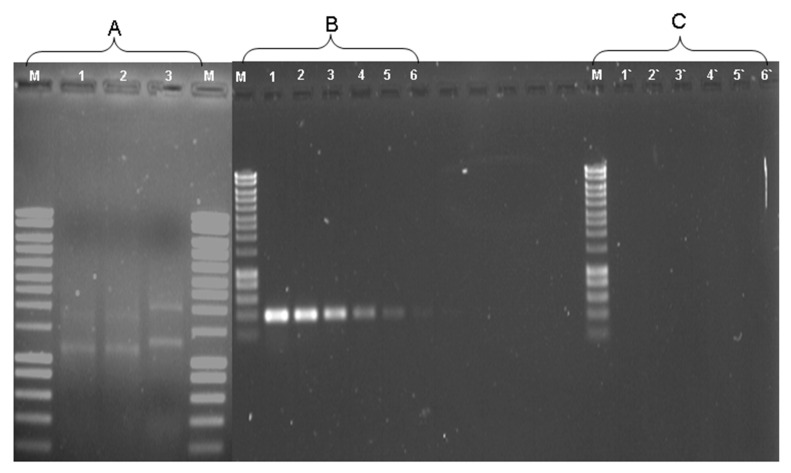
RNA extraction (**A**) from Chlorobenzene (lanes 1 and 2) and Fructose (lane 3). RT-PCR amplification of CBA-mRNA from *Alcanivorax* sp. strain HA03 grown on Chlorobenzene and on fructose was serially diluted (3.2-fold) with nuclease-free water, and 1 µL of each dilution was subjected to amplification by PCR. cDNA from Chlorobenzene (**B**) (Lanes 1–6) and from fructose (**C**) (lanes 1‘–6‘). M, molecular weight marker Hyperladder 1 (Bioline).

Chlorobenzene dioxygenase genes were characterized, which have the capability to express only in response to chlorobenzene. RT-PCR were applied using RNA extracted from *Alcanivorax* sp. strain HA03 growing on chlorobenzene or fructose (Figure 3A); the expected size was amplified only in the culture grown on chlorobenzene using FCbARNA (5′-GTGCGGCAGAAAGATAAAGC-3′) and RCbARNA (5′-GTCCCCGGTTTTAGGAATGT-3′) primers sets targeting CBA1 (α subunit) (Figure 3 Panel B-Lanes 1–6). No products were obtained either by using the extracted RNA from fructose-grown culture (Figure 3C) or in controls devoid of template cDNA or reverse transcriptase. To confirm CBA1 amplification by RT-PCR, sequencing of nearly 400 bp was identical to the gene fragment of CBA1 from genomic DNA of HA03. These results indicated the specific induction of CBA dioxygenase from HA03 in the presence of chlorobenzene.

### 3.2. Heterologous Expression of Chlorobenzene Dioxygenase Genes

The obtained ~4.5 kb fragment was digested using restriction enzymes (*EcoR*1 and *Pst*1) and cloned into pUC119 to give *pCB*, and transformed into *E. coli* JM109. To evaluate the transformation of chlorobenzene, *E. coli* JM109 harboring *pCB* was grown with IPTG at OD600 nm = 10. Whereas cells of *E. coli* JM109 harboring only pUC119 without insert showed that there is no chlorobenzene transformation and no metabolites were detected by HPLC analysis, cells of *E. coli* JM109 (*pCB*) had the capability of chlorobenzene transformation to 3 Chlorocatechol (3CC) with a rate of 1 µM/min, which was detected using HPLC with authentic standard from 3 Chlorocatechol (3CC) and showed an identical UV-absorption spectrum. The CBA1A2A3A4 genes from *Alcanivorax* sp. HA03 were confirmed for the functionalities of chlorobenzene dioxygenase by transformation and quantification of chlorobenzene into 3CC.

### 3.3. CBA Expression Analysis by SDS-PAGE

**Figure 4:**
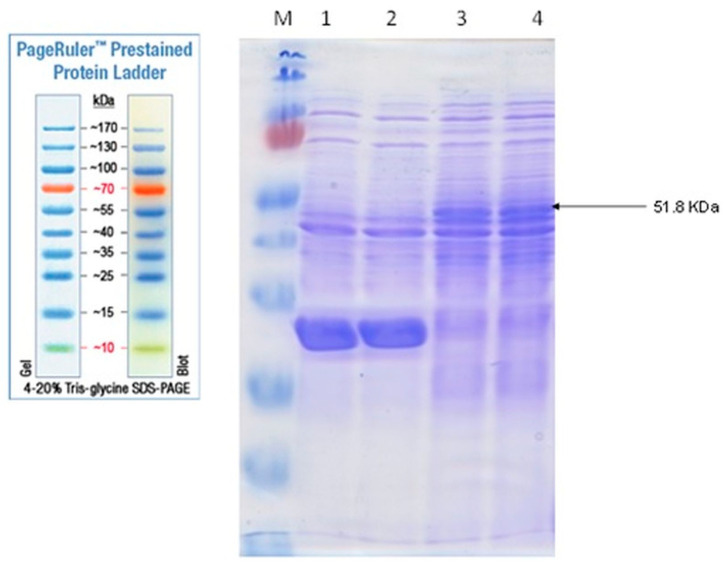
SDS-PAGE analysis of cell extracts of *E. coli* JM109 (lane 1), *E. coli* JM109 (pUC119) (lane 2), *E. coli* JM109 (*pCB*) pregrown in the presence of IPTG (lane 3) and *E. coli* JM109 (*pCB*) grown in the absence of IPTG (lane 4). In total, 10 µL of extracts were subjected to SDS-PAGE., M PageRulerTM Prestained protein ladder (Thermo Scientific).

The expression of CBA1 gene on pCB was analyzed by its translation to polypeptides at predicted sizes in *E. coli* JM109. The cloned *E. coli* cells, either cells harboring pUC119 or cells harboring *pCB*, were grown with and without IPTG. The comparisons between cell extracts were applied to SDS polyacrylamide gels, whereas the molecular weight of the denaturated protein subunits was determined. A prominent band at ~51.8 kDa was observed in cell extracts of the cloned *E. coli* cells harboring *pCB* (Figure 4), and was absent in *E. coli* cell extracts with only pUC119. This band is the expected molecular mass of the CBA1 gene product. The presence or absence of IPTG showed no effect on CBA expression.

### 3.4. PCR Amplification and Characterization of CC12Dos in Alcanivorax *sp.* HA03

**Figure 5:**
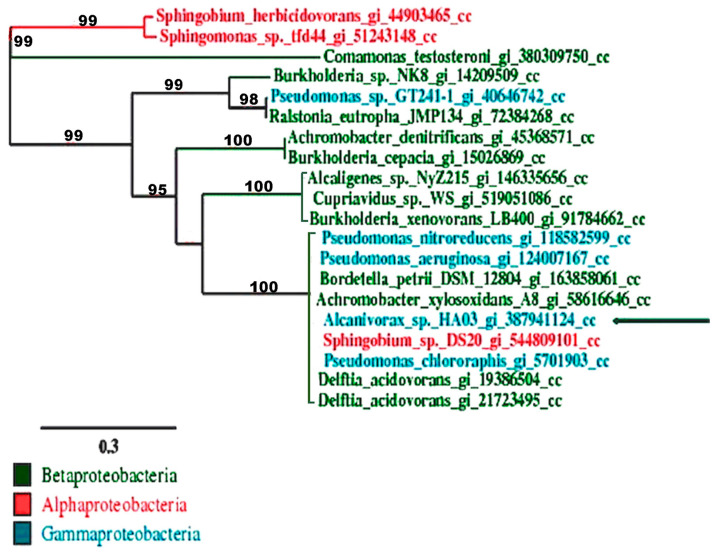
Phylogenetic tree for the chlorocatechol 1,2 dioxygenase from *Alcanivorax* sp. HA03 (indicated with an arrow) and its similarities with the other chlorocatechol dioxygenase from the other strains.

Few genes involved in the metabolism of chlorobenzene have been described in the literature; as 3-chlorocatechol is the bottleneck of the degradation chlorobenzene, it was attempted to identify genes responsible for the degradation of chlorocatechols by chlorocatechol pathways, which was initiated by the broad substrate specificity of chlorocatechol 1,2-dioxygenase. Sequence analysis of the insert using the PCR products obtained with the fw-cldioxmt and rev-cldioxmt primer set revealed the presence of the CC12Dos gene fragment. The analyzed PCR products were highly homologous (97%) to the gene fragment of the chlorocatechol 1,2-dioxygenase from *Delftia acidovorans*. The single read of the sequence obtained with fw-cldioxmt and rev-cldioxmt primer set exhibited a high similarity with the *tfdc* gene cluster of in *D. acidovorans* encoding chlorocatechol 1,2-dioxygenase. To amplify the compete gene sequence information, as mentioned above, of the gene putatively encoding the CC12Dos, a specific primer set (CC1,2HF (5′-ATGAACGAACGAGTGAAGCA-3′) and CC1,2HR 5′-CTGATGGCCTCGATCTTCAT-3′), using *Alcanivorax* sp. HA03 genomic DNA, was used as template. PCR product of the expected size 760bp was obtained, which was purified and sequenced using the same primers, and showed high similarity over the whole sequence with the tfdc gene of *D. acidovorans* with only four different nucleotides. The predicted amino acids of CC12Dos from *Alcanivorax* sp. HA03, which showed high similarity over the whole predicted amino acids from the tfdc gene of *D. acidovorans*, with only three different amino acids. The phylogenetic tree was obtained for the chlorocatechol 1,2 dioxygenase from *Alcanivorax* sp. HA03 (JQ687410), and chlorocatechol dioxygenase was obtained from other strains. It showed 99% similarity with the tfdc gene of *D. acidovorans* and 98% similarity with chlorocatechol 1,2-dioxygenase II from *Pseudomonas chlororaphis* RW71, which is the first microorganism shown to mineralize 1,2,3,4-tetrachlorobenzene via a 4,5-substituted chlorocatechol, (3,4,5,6-) tetrachlorocatechol (Figure 5).

### 3.5. Heterologous Expression of CC12Dos in E. coli JM109

**Figure 6:**
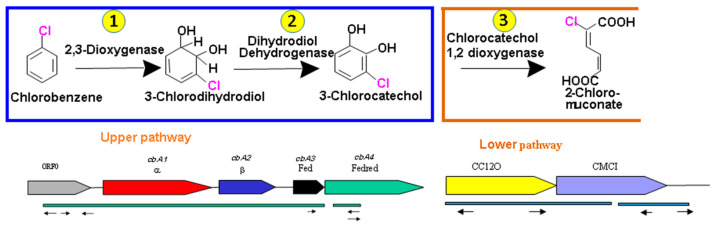
The catabolic pathway of chlorobenzene degradation by *Alcanivorax* sp. HA03. The catabolic enzyme’s (1) initial dioxygenase, (2) dihydrodiol dehydrogenase, (3) chlorocatechol 1,2 dioxygenase.

To analyze if the CC12Dos ORF from HA03 encode functional chlorocatechol 1,2-dioxygenase, the complete CC12Dos gene was amplified from DNA *Alcanivorax* sp. HA03 using primer set (CC1,2HF/CC1,2HR), cloned into the TA cloning kit pGEM^®^-T Easy Vector (Promega) and transformed by heat shock in *E. coli* JM109. *E. coli* JM109, harboring only the vector without insert, was applied as control. Screening of white colonies was done with colony PCR for half of the colonies, while the other half was left to reculture. The same primer set CC1,2HF/CC1,2HR was used for the amplification. The white colonies that harbored the inserted CC12Dos gene were recultured from the other half. The cloned *E. coli* cells were grown with IPTG, where the transformation of 3-Chlorocatechol (200 μM) was tested by resting cells of an OD600 nm of up to 10. The activity was observed against 3-Chlorocatechol, and after 18 h of incubation, approximately 12 μM of 2-chloromucounate was produced due to 3-Chlorocatechol transformation, as evidenced by the HPLC analysis and comparison with an authentic standard. This is in agreement with the postulated pathway (Figure 6).

### 3.6. CC12Dos Expression Analysis by SDS-PAGE

**Figure 7:**
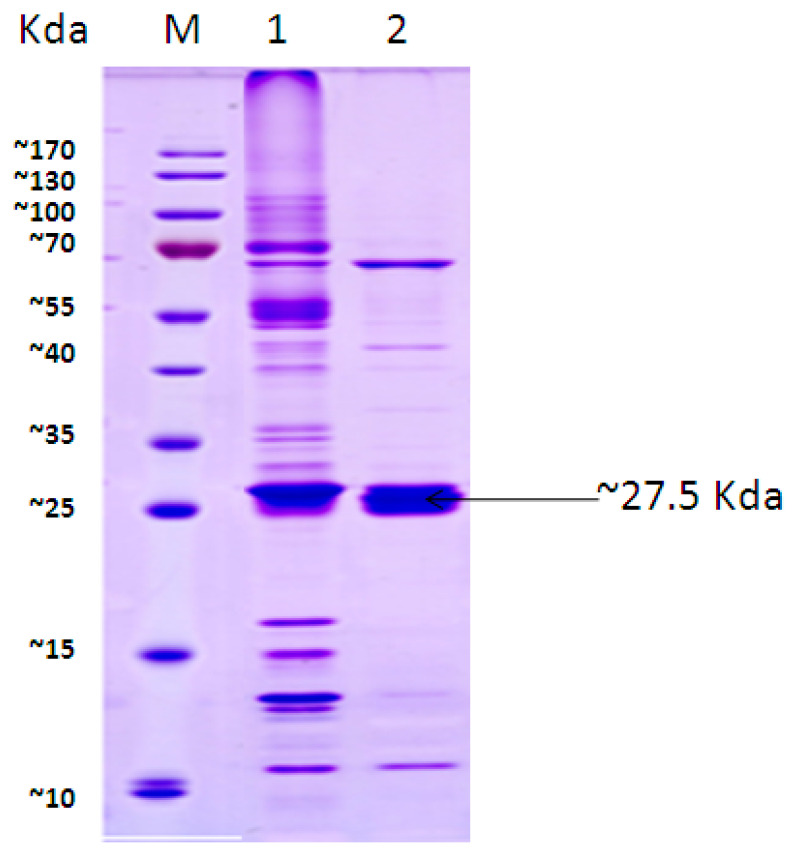
SDS-PAGE analysis of cell extracts of *E. coli* JM109 with CC12Dos gene insert diluted 1:1 (lane 1) and 1:10 (lane 2); M PageRuler™ Prestained 10–170 kDa (Thermo Scientific).

To check the molecular weight and the efficiency of chlorocatechol 1,2-dioxygenase (CC12Dos), SDS-PAGE was used to determine the expression of CC12Dos and its translation to polypeptides with the predicted sizes. CC12Dos gene was cloned in TA vector (pGEM^®^-T Easy Vector) and transformed by heat shock in *E. coli*. The cloned cell extracts harboring the vector and the inserted CC12Dos gene were diluted using 2X treatment buffer into 1:1 and 1:10 and compared by SDS-PAGE. A prominent band with a molecular mass of approximately 27.5 kDa was observed in cell extracts of the cloned *E. coli*; overexpression was present after protein dilution from 1:1 to 1:10 (Figure 7).

## 4. Discussion

In the last few decades, due to agricultural and industrial practices, many hydrocarbons were inserted into the environment. This has led to the accumulation of many aromatic and chloroaromatic compounds. The topic of haloalkaliphiles and its degradation or transformation for organic pollutants has not been adequately studied; there are few studies related to the degradation of aromatic and chloroaromatic pollutants and its metabolic pathways by this group of haloalkaliphiles. Most of the information on aromatic compounds degradation in extreme saline and alkaline environments was obtained on an ecological scale, and very few studies have been performed to demystify the metabolic pathways for the degradation of aromatic pollutants in extreme environments. On the other hand, little is known about the biodegradation of chlorobenzene in extreme salinity and alkalinity environments. The novel halophilic alkaliphilc *Alcanivorax* sp. HA03 was isolated from soda lakes in Wadi El Natrun, which has the capability to degrade benzene, toluene and chlorobenzene (CB) [14].

Biodegradation of CB under normal aerobic conditions has been previously described for several bacterial species, such as *Alcaligenes, Pseudomonas, Xanthobacter*, *Rhodococcus*, *Ralstonia*, *Acidovorax*, *Planococcusa* and *Burkholderia* [6,12,35,36,37]. Only two reported strains, *Alcanivorax* sp. HA03 and *Planococcus* sp. strain ZD22, have the ability to degrade CB at salinity conditions and are considered haloalkaliphile (Li et al., 2006; Hassan et al., 2012) [13,14]. The degradation of CB by those strains under extreme conditions expands their metabolic capabilities.

In the upper pathway, degradation of CB is initiated by ring-hydroxylation of CB, forming chlorinated dihydrodiols. The dihydrodiols are then subjected to further dehydrogenation, yielding corresponding chlorocatechols (Figure 6). In the lower pathway, chlorocatechols was ring cleaved using ring cleavage enzymes and a subsequent reaction that results in the elimination of chloride (Figure 6). The degradation of halobenzene is similar to the degradation of benzene, toluene and ethylbenzene in the initial steps [32,33]. Since the α subunit plays an important role as a catalytic component in the oxygenase for substrate specificity degradation [38,39,40], based on the catalytic activity of oxygenase, which is mainly based on the α subunit, these enzymes were classified based on the α subunit sequence. The oxygenase classification comparisons confirmed that the grouping of the oxygenases largely correlates with the respective substrate specificity [41,42]. Each subunit in these enzymes contains a Rieske [2Fe–2S] cluster and mononuclear iron [43], which could be the site of dioxygenase activity and catalysis.

Our results showed that CBA1 exhibited a higher amino acid sequence identity, i.e., 99%, with a halobenzene-degrading bacterium, *Burkholderia fungorum* FLU100 [32,33], and 92% with *Psudeumonus* -BTEX degrading species (Figure 2). This indicates that CBA1 from HA03 has an amino acid sequence compatible with the benzene, halobenzene and toluene dioxygenase (CBO) system. The complete degradation of CB by HA03 is probably related to the presence of the CBO system.

The degradation of chlorinated aromatic compounds by aerobic bacteria is usually followed by the formation of chlorinated catechol as an intermediate [6], as the 3-chlorocatechol is the bottleneck of the complete mineralization of chlorobenzene. In this study, chlorocatechol 1, 2 dioxygenase gene (CC12Dos) was amplified and identified. Phylogentic analysis of CC12Dos with the other similar chlorocatechol dioxygenase (Figure 5) showed 99% similarity, with a difference of only two amino acids compared to chlorocatechol 1,2 dioxygenase from *Delftia acidovorans* P4a, which is an alkalitolerant strain isolated from highly alkalinity sites polluted with organochlorines, such as the investigated strain in this study *Alcanivorax* sp. HA03. *Delftia acidovorans* P4a has the capability to complete the metabolic pathway degradation of 2,4-dichlorophenoxyacetic acid and 2-methyl-4-chlorophenoxyacetic acid under alkaline conditions [44]. CC1,2O from HAo3 showed 98% similarity, with a difference of only three amino acids compared to the chlorocatechol 1,2-dioxygenase gene *tetC* from *Pseudomonas chlororaphis* RW71 (Potrawfke et al., 2001) [45], where *Pseudomonas chlororaphis* RW71 is the first microorganism shown to mineralize 1,2,3,4-tetrachlorobenzene via a 4,5-substituted chlorocatechol [46]. CC12Dos showed 98% similarity with chlorocatechol 1,2-dioxygenase gene from *Achromobacter xylosoxidans* A8 isolated in the Czech Republic from soil contaminated with PCB [47], and has the capability to mineralize 2-CB and 2,5-DCB. *A*. *xylosoxidans* A8 harbors two plasmids, pA81 (98.2 kbp) and pA82 (ca. 250 kbp), the former of which encodes for CB degradation [48]. The high similarity of CC1,2O from *Alcanivorax* sp. HA03 with the other cholorocatechol 1,2 dioxygenase from strains isolated from extreme environment either heavily contaminated with organochlorines or highly alkaline conditions in an aqueous environment. This indicated that in this type of gene, CC12Dos may be found only in extremophiles strains, or these extremophiles strains had plasmid encoding for extremophiles and chloroaromatic degradation.

## 5. Conclusions

The results indicate that the Haloalkaliphilic *Alcanivorax* sp. HA03 is capable of mineralizing chlorobenzene (CB). This capability is probably related to the presence of the specific chlorobenzene dioxygenase cluster CBA1A2A3A4 in this strain, which shows high homology with other similar dioxygenase described for CB, BTEX and biphenyl aromatic compounds. We propose that CBA1 could be specific for the CB degradation assayed in this study. This study characterized the CBA1 protein and obtained evidence on its expression only in the presence of CB and its function in the biodegradation of CBs. The lower metabolic pathway of CB was detected through intradiol ring cleavage of chlorocatechol by amplification and characterization of genes encoding a chlorocatechol 1,2 dioxygenase (CC12DOs) in *Alcanivorax* sp. HA03 transformed into chloromuconate.

To the best of our knowledge, very little is known about haloalkaliphilic CB degraders for either metabolic pathways or phylogenetic diversity or their CB-degrading enzymes. Isolation and characterization of chlorobenzene bacterial degraders and also the novel chlorobenzene dioxygenase gene cluster and chlorocatechol 1,2-dioxygenase (CC12Dos) under haloalkaliphilic condition are very interesting for the bioremediation of this highly toxic compound under extreme salinity and alkalinity conditions.

## Data Availability

Not applicable.
